# Continuity of Care in General Practice in Australia: A Whole‐Of‐Population Serial Cross‐Sectional Study

**DOI:** 10.5694/mja2.70229

**Published:** 2026-06-17

**Authors:** Jennifer Welsh, Rachel Freeman‐Robinson, Danielle C. Butler, Rachael Eddowes, Amelia Yazidjoglou, Angus Douglas, Kirsty A. Douglas, Nina Lazarevic, Hsei‐Di Law, Grace Joshy, Susan Trevenar, Tsheten Tsheten, Adrian Webster, Michael Frost, Bernice Cropper, Sally Hall Dykgraaf, Christine B. Phillips, Emily Banks, Rosemary J. Korda

**Affiliations:** ^1^ National Centre for Epidemiology and Population Health Australian National University Canberra Australian Capital Territory Australia; ^2^ Institute for Urban Indigenous Health Windsor Queensland Australia; ^3^ School of Medicine and Psychology Australian National University Canberra Australian Capital Territory Australia; ^4^ Australian Institute of Health and Welfare Canberra Australian Capital Territory Australia

**Keywords:** continuity of patient care, general practice, health policy, healthcare disparities

## Abstract

**Objective:**

To quantify continuity of care in general practice in the Australian population, including variation according to patient characteristics and over time, to support ongoing policy reforms directed towards improving general practice care.

**Design and Setting:**

Repeated cross‐sectional analyses of linked whole‐of‐population data from the Medicare Benefits Schedule, the Medicare Consumer Directory and the Census of Population and Housing (2021).

**Participants:**

Continuity was assessed in people with at least four general practitioner visits in a 2‐year period (about 80% of the population).

**Main Outcome Measure:**

Relational continuity of care in general practice, measured with the Usual Provider Index, for eight overlapping 2‐year periods (2016–2017 to 2022–2023). High continuity was defined as having ≥ 70% of visits with one provider.

**Results:**

About one‐third of the population had high continuity of care (range: 31.3% in 2018–2019 to 37.2% in 2020–2021). After adjustment for age, sex and remoteness, high continuity was more common among those with greater care needs, including those who were older (≥ 70 years vs. 0–14 years: adjusted prevalence ratio [aPR], 1.88) or with health conditions (e.g., ≥ 3 vs. none: aPR, 1.14) and those who were living in more disadvantaged areas (e.g., most vs. least disadvantaged: aPR, 1.22), born overseas (e.g., born in Southern or Eastern Europe vs. born in Australia or New Zealand: aPR, 1.20) or not proficient in English (aPR, 1.29). However, it was less common for females compared with males (aPR, 0.90) and those living remotely (e.g., very remote vs. major cities: aPR, 0.43).

**Conclusion:**

While most people in Australia do not receive continuous care in general practice with a specific provider, those with greater healthcare needs are more likely to. With ongoing policy reforms, monitoring continuity of care may provide insights into the consequences for quality of care.

## Plain Language Summary

**The Known:**

Continuity of care is central to quality primary care, but data for the general Australian population are lacking.

**The New:**

Among the approximately 80% of Australians with at least four general practitioner visits in a 2‐year period, around one‐third had high continuity (≥ 70% of visits with their usual general practitioner), with small increases since 2020. Proportions were higher among those with greater healthcare needs (e.g., older age, more socioeconomically disadvantaged, with health conditions), but lower in remote areas.

**The Implications:**

Monitoring continuity may provide insights into consequences of health policy reforms, both intended and unintended, to optimise primary care in Australia.

## 1 Introduction

Primary care is foundational to Australia's and other health systems and plays a critical role promoting health and health equity in the population [1]. It is designed as the first point of access to the Australian healthcare system and aims to provide ongoing, coordinated care throughout the life course [2].

A central tenet of quality primary care is continuity of care (CoC), which refers to an ongoing patient–provider relationship that extends beyond a specific episode of care [3, 4, 5]. There are three types of CoC: informational (using information from past events to inform current care), management (consistent approach to managing conditions) and relational (an ongoing patient–provider relationship) [4]. The benefits of continuity are well established: CoC has been shown to facilitate trust between patients and their general practitioner, improve coordination of services and is associated with higher patient satisfaction [3, 6, 7], lower costs to the patient and health system [6, 8, 9], and better health outcomes, including improved continuation with medications, and lower hospital admission and mortality rates [10, 11, 12, 13]. As a result, CoC is recognised as critical component of care in general practice settings [14].

Despite its importance, very little is known about levels of CoC in general practice in the Australian population. Existing Australian evidence is limited to clinical populations (e.g., people with diabetes [15]) and/or older populations [12, 16] and as such, basic but critical information on CoC remains largely unknown. This includes information on overall levels, which patient groups are least likely to receive continuous care and how CoC has changed over time. This evidence is important, particularly in the context of ongoing policy reform.

Several policy initiatives have been implemented to improve general practice care in recent years. This includes MyMedicare, which aims specifically to improve CoC (among other things) by offering incentives for patients to register with their preferred general practice and provider [17], as well as a range of other reforms designed to improve availability (e.g., urgent care clinics, telehealth) and affordability (e.g., bulk billing initiatives) of services. Each reform has the potential to either support or unintentionally undermine CoC, with ramifications for the long‐term success of the policy to support good health outcomes.

In this study, we aimed to use whole‐of‐population data to quantify CoC in general practice in Australia, including variation in CoC according to patient characteristics and over time.

## 2 Methods

We used Medicare data (Medicare Consumer Directory and Medicare Benefits Schedule [MBS] claims data, 2017–2023) linked to the 2021 Census of Population and Housing, available through the Person Level Integrated Data Asset [18]. The MBS data contains information on all claims for medical services that are reimbursed under Medicare, which covers all Australian citizens and permanent residents. Each claim contains an MBS item number (indicating type of visit) and date of service, and de‐identified Medicare patient and provider numbers to distinguish unique patients and doctors. The 2021 Census included usual residents of Australia on the night of 10 August 2021 living in private and non‐private dwellings, with an estimated 95.8% person response rate [19].

Our primary outcome was relational CoC in general practice, measured with the Usual Provider Index (UPI) and derived from MBS data, which measures the proportion of general practitioner visits provided by an individual's main provider [20]. Given that proportions are sensitive to the number of services received, and to avoid artificially large fluctuations in scores over time, we derived UPI scores for individuals with at least four visits to a general practitioner in a 2‐year period, consistent with previous research [20]. As such, our study population included those who had at least four general practitioner visits (details in Table S1) in the (overlapping) 2‐year study periods between 2016–2017 and 2022–2023.

For each 2‐year period, we described the proportion of the population with high CoC, defined as a UPI score of ≥ 0.70 (i.e., minimum of 70% of visits with their usual provider), consistent with previous research [21]. For the most recent period of data (2022–2023), we also described CoC proportions separately by age, sex, remoteness and area‐level disadvantage.

To more extensively examine patient variation in CoC, we restricted the study population to those linked to a 2021 Census record and included a larger range of sociodemographic and health characteristics recorded on the Census. This included relationship status, employment status, education level, country of birth, English proficiency, annual household income and presence (and count) of selected chronic health conditions. To quantify associations between these characteristics and our binary outcome (UPI ≥ 0.70 vs. < 0.70), we estimated prevalence ratios and 95% confidence intervals using Poisson regression with robust standard errors [22]. For each characteristic (separately), we used three models: model 1 was unadjusted, and we then cumulatively adjusted for sex and 5‐year age group (from 0 to 4 years to 80 years and older, model 2) and remoteness (model 3). Associations between sociodemographic characteristics and high CoC were further adjusted for presence of the specific health conditions (none compared with one or more, for model 4).

In supplementary analyses, we quantified CoC for the Medicare population using alternative cut‐points to define high CoC (0.75 and 0.80) to assess whether the findings were sensitive to different definitions. Data were analysed using Stata Version 18 and SAS 9.4. Ethics approval for this study was granted by the Australian National University Human Research Ethics Committee (HREC 2021/619).

## 3 Results

There were 24.7 million people with at least one out‐of‐hospital MBS claim (Medicare population) in 2022–2023. Of these, approximately 79% (19.4 million) had at least four general practitioner visits and were included in the study. Similarly, approximately 77% (17.3 million out of 22.5 million) of the eligible Census population had at least four general practitioner visits (Figure S1). The proportions of the Medicare population with at least four general practitioner visits were similar across study periods (range, 80.4% in 2018–2019 and 2019–2020, 78.4% in 2020–2021 and 2021–2022). Females, older people and people living in cities were the most likely to be eligible for a UPI score (Table S2).

**FIGURE 1:**
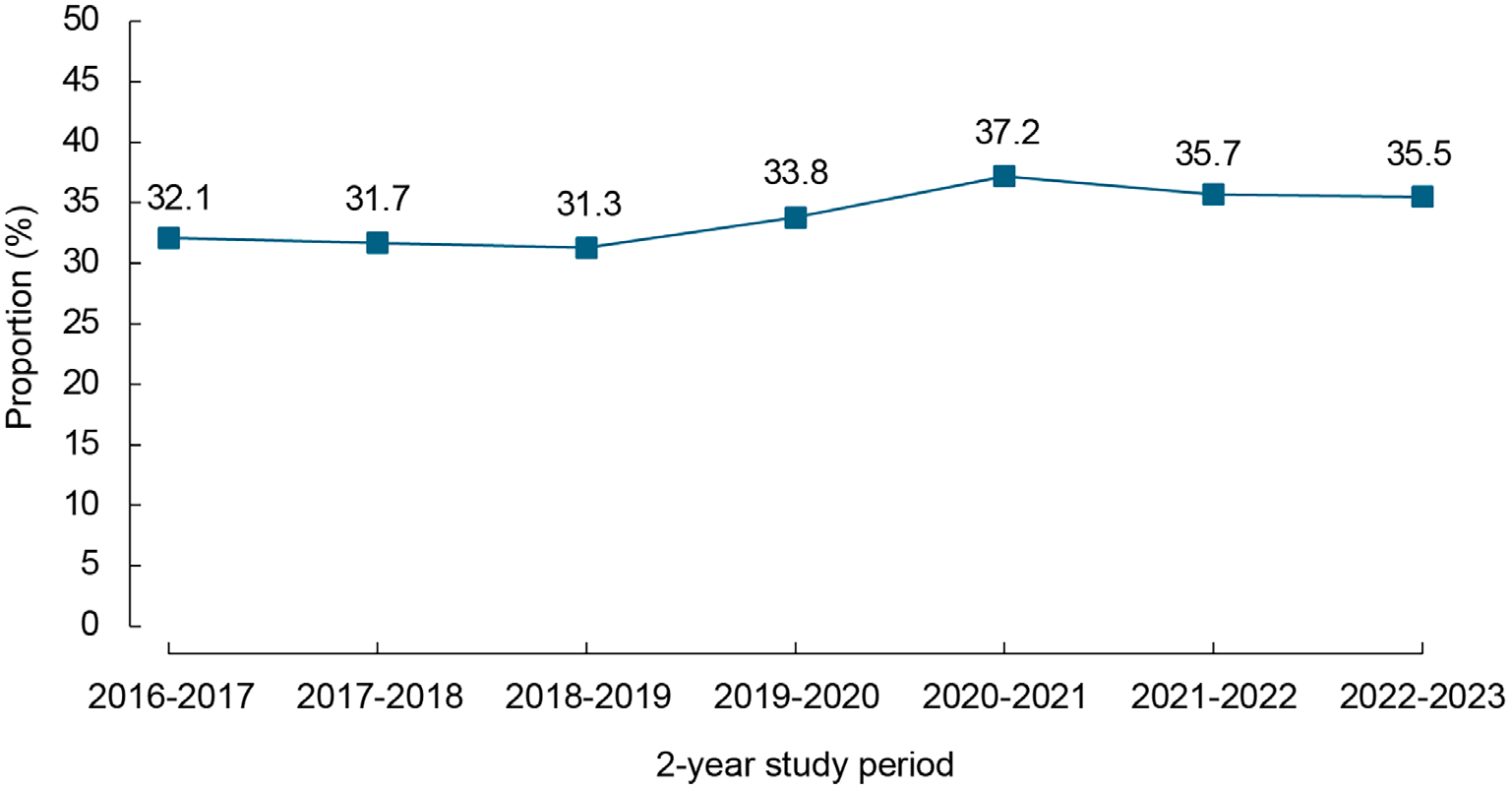
Proportion of the eligible Medicare population with high continuity of care (UPI ≥ 0.70) in each 2‐year period from 2016–2017 to 2022–2023.^a^ UPI, Usual Provider Index. ^a^A UPI of 0.70 means that at least 70% of general practitioner visits were provided by an individual's main (i.e., most frequent) provider.

In 2022–2023, 35.5% of the eligible Medicare population had high CoC. Before 2020, 31.3%–32.1% had high CoC, with proportions increasing from 2020 onwards, peaking in 2020–2021 at 37.2% (Figure 1). Proportions with high CoC were slightly higher for males compared with females, increased with age (0–14 years, 22.5%; ≥ 70 years, 53.5%) and area‐level socio‐economic disadvantage (least disadvantaged, 33.4%; most disadvantaged, 39.1%), and decreased with remoteness (major cities: 36.3%; very remote: 15.3%) (Table 1). Proportions with high CoC were similar for those with < 20 general practitioner visits (33.3%–34.6%) but were higher among those with 20–39 (39.6%) and ≥ 40 (46.3%) visits. Variation in high CoC between patient groups and over time was materially unchanged when using different cut‐points to define high CoC (Table S3).

**TABLE 1:** Number and proportion (%) of the Medicare study population with high continuity of care (UPI ≥ 0.70) by patient characteristics, 2022–2023.^a^

	Medicare study population (number with ≥ 4 general practitioner visits in 2022–2023)	High continuity of care, number (%)
Total	19,430,000	6,905,392 (35.5%)
Sex		
Male	8,857,404	3,332,477 (37.6%)
Female	10,572,596	3,572,915 (33.8%)
Age group (years)^b^		
0–14	3,005,408	672,091 (22.4%)
15–24	1,979,643	477,637 (24.1%)
25–44	5,111,869	1,459,107 (28.5%)
45–69	6,427,349	2,742,667 (42.7%)
≥ 70	2,905,731	1,553,890 (53.5%)
Remoteness area^c^		
Major cities	13,764,417	4,996,460 (36.3%)
Inner regional	3,217,967	1,112,430 (34.6%)
Outer regional	1,329,902	439,923 (33.1%)
Remote	153,370	41,298 (26.9%)
Very remote	63,581	9701 (15.3%)
Other	900,763	305,580 (33.9%)
SEIFA IRSD^d^		
Q1 (most disadvantaged)	3,640,948	1,423,050 (39.1%)
Q2	3,667,811	1,336,274 (36.4%)
Q3	3,730,034	1,308,052 (35.1%)
Q4	3,749,781	1,280,538 (34.1%)
Q5 (least disadvantaged)	3,711,255	1,239,685 (33.4%)
Missing	930,171	317,793 (34.2%)
Number of general practitioner visits		
4–6 visits	4,220,352	1,423,149 (33.7%)
7–9 visits	3,551,317	1,182,800 (33.3%)
10–14 visits	4,278,124	1,446,941 (33.8%)
15–19 visits	2,668,802	923,726 (34.6%)
20–39 visits	3,755,208	1,485,971 (39.6%)
≥ 40 visits	956,197	442,805 (46.3%)

Abbreviations: IRSD, Index of Relative Socio‐Economic Disadvantage; SEIFA, Socio‐Economic Indexes for Areas; UPI, Usual Provider Index.

^a^ A UPI of 0.70 means that at least 70% of general practitioner visits were provided by an individual's main (i.e., most frequent) provider.

^b^ Age at 1 January at the start of the 2‐year period, ascertained from the Medicare Consumer Directory.

^c^ Remoteness area based on the Statistical Area Level 1 of the individual's usual residence derived from the Medicare Consumer Directory (https://www.abs.gov.au/statistics/standards/australian‐statistical‐geography‐standard‐asgs‐edition‐3).

^d^ SEIFA IRSD in population‐based quintiles, from Q1 (most disadvantaged) to Q5 (least disadvantaged), based on the Statistical Area Level 1 information of the individual's usual place of residence (https://www.abs.gov.au/websitedbs/censushome.nsf/home/seifa).

**FIGURE 2:**
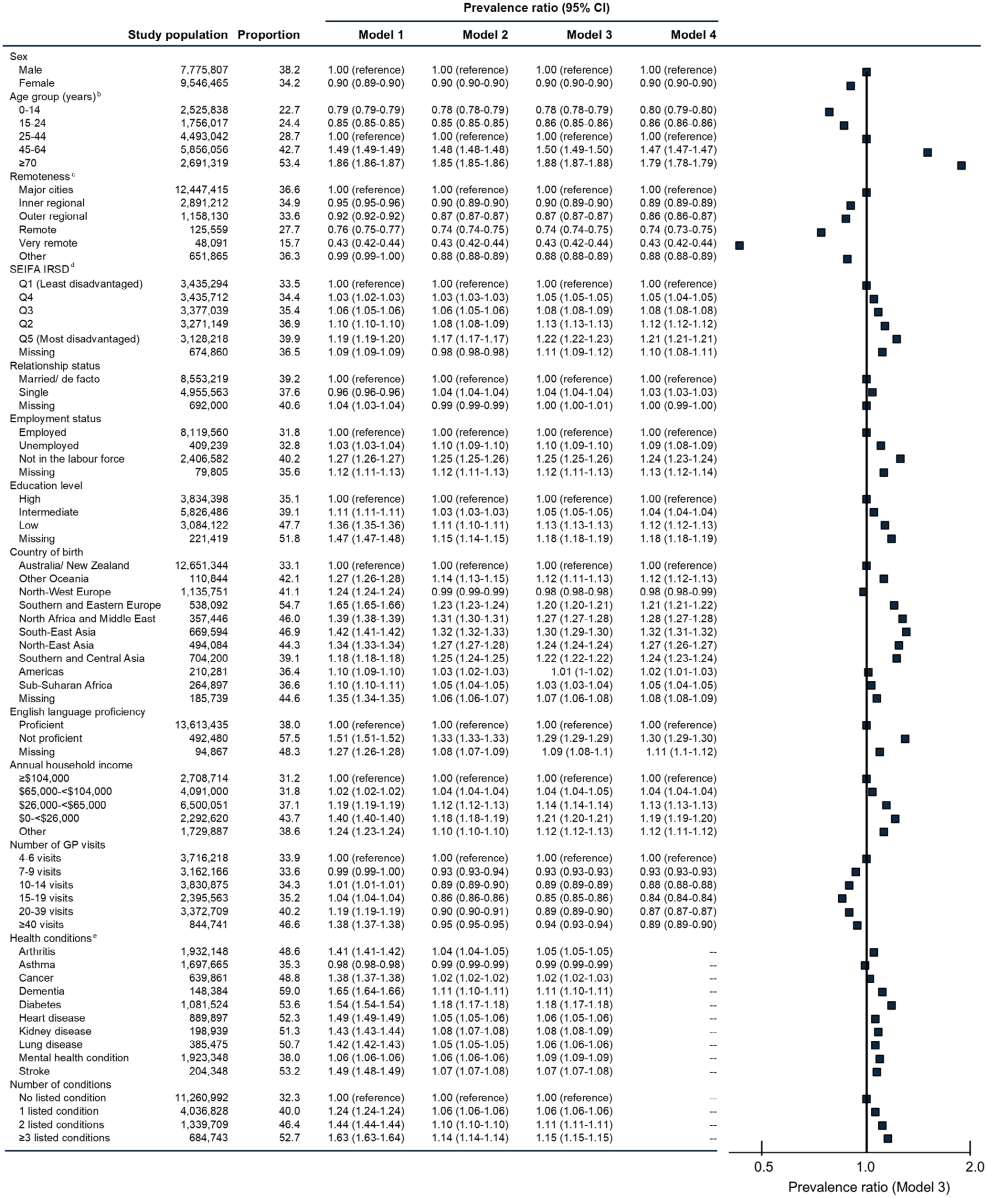
Proportion of the eligible Census 2021 population with high continuity of care (UPI ≥ 0.70) and prevalence ratios with 95% confidence intervals describing the association between high continuity and socio‐demographic and health characteristics, 2022–2023.^a^ IRSD, Index of Relative Socio‐Economic Disadvantage; SEIFA, Socio‐Economic Indexes for Areas; UPI, Usual Provider Index. ^a^Model 1 is unadjusted; Model 2 is adjusted for sex and 5‐year age group (from 0 to 4 years to 80 years and older); Model 3 is further adjusted remoteness; Model 4 is further adjusted for presence of a health condition. The figure is plotted on a log scale. Eligible population are those with ≥ 4 visits to a general practitioner in 2022–2023. A UPI of 0.70 means that at least 70% of general practitioner visits were provided by an individual's main (i.e., most frequent) provider. Variables were ascertained from the 2021 Census, except for sex and age (ascertained from Medicare Consumer Directory) and frequency of general practitioner visits (derived from Medicare Benefits Schedule claims data). ^b^Age group is based on age at 1 January in each study period. Results for the following variables are restricted by age, as measured at the time of Census: English language proficiency and marital status restricted to people aged ≥ 18 years; employment status restricted to people aged 15–64 years; and education level restricted to people aged ≥ 25 years. ^c^Remoteness area based on the Statistical Area Level 1 of the individual's usual place of residence (https://www.abs.gov.au/statistics/standards/australian‐statistical‐geography‐standard‐asgs‐edition‐3). ^d^SEIFA IRSD in population‐based quintiles, from Q1 (most disadvantaged) to Q5 (least disadvantaged), based on Statistical Area Level 1 information on the individual's usual place of residence (https://www.abs.gov.au/websitedbs/censushome.nsf/home/seifa). ^e^Health conditions: Self‐reported from list of 10 conditions collected as part of the 2021 Census. Cancer includes remission; dementia includes Alzheimer's disease; diabetes excludes gestational diabetes; heart disease includes heart attack or angina; lung disease includes chronic obstructive pulmonary disease or emphysema; mental health condition includes depression or anxiety.

Among the Census population in 2022–2023, sociodemographic differences in high CoC were similar to those of the Medicare population and were notably high among people from Southern and Eastern Europe (54.7%), not proficient in English (57.5%), with chronic health conditions (> 45% for eight out of ten of the measured conditions) and with more health conditions (none, 32.3%; one condition, 40.0%; two conditions, 46.4%; three or more conditions, 52.7%) (Figure 2). After adjustment for age, sex and remoteness, high CoC was more common among those who were older (e.g., ≥ 70 years vs. 0–14 years: adjusted prevalence ratio [aPR], 1.88), not in the labour force (aPR, 1.25) or unemployed compared with those who were employed (aPR, 1.10), living in more disadvantaged areas (most vs. least disadvantaged: aPR, 1.22), with lower levels of education (low vs. high: aPR, 1.13) or lower annual household income (e.g., < $26,000 vs. ≥ $104,000: aPR, 1.21), born overseas (e.g., born in Southern or Eastern Europe vs. born in Australia or New Zealand: aPR, 1.20) and not proficient in English compared with those who were (aPR, 1.29). Proportions were relatively high for people with chronic conditions: compared with none of the listed conditions, aPRs were 1.06, 1.11 and 1.15 for those with one, two or three or more of the conditions (Figure 1). High CoC was less common among females compared with males (aPR, 0.90) and those living in more remote areas (e.g., very remote vs. major cities: aPR, 0.43). Further adjustment for presence of a specified health condition did not materially change the relationships between sociodemographic characteristics and high CoC (Figure 1, model 4).

## 4 Discussion

Around one‐third of the Australian population who visit a general practitioner at least four times in any 2‐year period have high CoC, meaning they see the same provider for at least 70% of these visits. The proportion of this population with high continuity was relatively stable before 2020, and was around four to six percentage points higher in each of the 2‐year periods from 2020–2021 to 2022–2023.

Proportions with high CoC were greater for population groups with greater healthcare needs, including those of older age, with chronic health conditions or who were more socioeconomically disadvantaged. This finding is consistent with previous evidence from Australia and New Zealand on clinical populations that showed that CoC is higher among older patients [23], people of lower socio‐economic status [24, 25] and those with higher healthcare needs [24], including those with chronic health conditions and in poorer health [23]. However, even among these groups, at most around half of the eligible Australian population had high CoC. Further, a large majority of people living remotely did not have high CoC, also consistent with earlier research [25].

That around two‐thirds of the eligible population did not have high CoC indicates potential for improving quality of primary care in Australia. While the underlying reasons for low continuity could not be assessed with administrative data, these are likely explained in part by access issues. The majority of Australians (78.7% in the 2023–2024 financial year) had a preferred general practitioner, yet around one‐third (33.6%) reported not always being able to see them when needed, with appointment wait times and costs being key barriers [26]. Low CoC in rural areas likely reflects the higher turnover of doctors in rural and remote areas [27]. Thus, policies that address access issues—such as recent incentives to support all general practitioners to bulk bill [28] and the recognition of rural generalists as an official general practice specialty [29]—may support CoC with a preferred general practitioner. Importantly however, low continuity does not necessarily reflect lack of access. For example, lower continuity could reflect patients seeing multiple providers within the same practice, or multiple providers at locations close to home and work.

Proportions with high CoC were relatively stable before 2020 but increased by around five percentage points from 2020. This increase may reflect high uptake of Medicare‐funded telehealth services [30]. These services were substantially expanded in March 2020 in response to the COVID‐19 pandemic, and were conditional on an established patient–provider relationship [31]. While it is unclear whether this increase can be directly attributed to telehealth, it highlights how monitoring CoC could be used to provide insights into the consequences of health policies.

Continuity of care is an explicit policy goal outlined by the Strengthening Medicare Taskforce and Australia's Primary Health Care 10 Year Plan 2022–2032 [14, 32]. However, government policies operationalise continuity at the practice level rather than provider level, as exemplified by MyMedicare. This approach reflects that care delivered by multiple practitioners from a single practice is likely to facilitate informational and management continuity in a way that promotes flexible provision of care [33], noting that there is uncertainty as to whether practice‐level CoC provides the same benefits as provider‐level continuity [34]. As such, contemporary monitoring capability is substantially limited by the fact that practice identifiers are not currently available within MBS data, although there have been efforts to create proxy identifiers [35]. While other data sources contain practice identifiers (e.g., Lumos [36], which contains linked health service data from NSW), including such identifiers in MBS data as part of existing national data assets would enable whole‐of‐population monitoring of CoC in a way that is more directly tied to policy goals and objectives.

This study provides the first whole‐of‐population evidence on CoC in general practice in Australia, including the distribution of continuity across patient groups. However, our findings should be interpreted with limitations in mind. The UPI is a widely used indicator of individual provider relational CoC but does not directly assess trusted patient–provider relationships. The UPI also does not measure continuous care delivered across multiple practitioners within a single practice, that is, informational CoC or management CoC [4, 37]. Nevertheless, while the current UPI is an imperfect measure, it is likely to be useful in providing evidence on whether primary healthcare policies have improved, or inadvertently compromised, provider‐specific CoC.

## 5 Conclusion

Continuity is a component of quality primary care and is recognised as an important policy goal, particularly as the population ages and experiences increasingly complex chronic health concerns [38]. Evidence from this study indicates that while the distribution of CoC is to some extent pro‐equity in Australia in that those most in need are the most likely to have CoC, most people do not receive continuous general practice care from the same provider. In the face of ongoing pressures and policy reform directed towards general practice in Australia, there are likely to be benefits of ongoing monitoring of population levels of CoC to provide insights into the broader consequences of general practice reforms, both intended and unintended.

## Author Contributions

Emily Banks: conceptualisation, methodology, funding acquisition. Danielle C. Butler: funding acquisition, conceptualisation, methodology, writing – review and editing. Bernice Cropper: writing – review and editing. Angus Douglas: writing – review and editing. Kirsty A. Douglas: writing – review and editing, funding acquisition. Rachael Eddowes: writing – original draft. Rachel Freeman‐Robinson: writing – original draft. Michael Frost: Writing – review and editing. Sally Hall Dykgraaf: writing – review and editing, funding acquisition. Grace Joshy: writing – review and editing, funding acquisition. Rosemary J. Korda: Conceptualisation, methodology, writing – original draft, funding acquisition. Hsei‐Di Law: formal analysis, writing – review and editing. Nina Lazarevic: formal analysis, writing – review and editing. Christine B. Phillips: funding acquisition, writing – review and editing. Susan Trevenar: writing – review and editing. Tsheten Tsheten: writing – review and editing. Adrian Webster: writing – review and editing. Jennifer Welsh: conceptualisation, methodology, formal analysis, writing – original draft, funding acquisition. Amelia Yazidjoglou: writing – review and editing.

## Funding

This project was funded by the Australian Institute of Health and Welfare and the Australian Government's Medical Research Future Fund grant (grant number 2006309). Emily Banks receives support from the National Health and Medical Research Council (grant number 1136128).

## Disclosure

Not commissioned; externally peer reviewed.

## Conflicts of Interest

This project was partly funded by the Australian Institute of Health and Welfare (AIHW), an independent government agency with responsibility for national reporting of health and welfare statistics. Adrian Webster, Bernice Cropper and Michael Frost are employed at the AIHW and had input in drafting and reviewing the manuscript, specifically regarding the interpretation of the findings in the broader context of the Australian healthcare system. The other authors declare that they had no competing interests.

## Supporting information


**Table S1:** MBS codes used to identify general practitioner visits.
**Table S2:** Proportion (%) of the Medicare study population with at least 4 general practitioner visits (UPI coverage), 2016–2017 to 2022–2023.
**Table S3:** Proportion (%) of the eligible Medicare study population with high continuity of care using different UPI cut points, by patient characteristics 2016–2017 to 2022–2023.
**Figure S1:** Study flow diagram for the Census study population for the 2022‐23 study period.
STROBE Statement—checklist of items that should be included in reports of observational studies.

## Data Availability

Data from the Person Level Integrated Data Asset are available for approved projects to approved government and non‐government users.
