# Supplementary material for: Continuity of Care in General Practice in Australia: A Whole‐Of‐Population Serial Cross‐Sectional Study
Source: Med J Aust. 2026 Jun 17;224(6):e70229. doi: 10.5694/mja2.70229 (PMC13275638; doi:10.5694/mja2.70229)
Supplement: Supplementary file 1 — Table S1: MBS codes used to identify general practitioner visits. Table S2: Proportion (%) of the Medicare study population with at least 4 general practitioner visits (UPI coverage), 2016–2017 to 2022–2023. Table S3: Proportion (%) of the eligible Medicare study population with high continuity of care using different UPI cut points, by patient characteristics 2016–2017 to 2022–2023. Figure S1: Study flow diagram for the Census study population for the 2022‐23 study period. STROBE Statement—checklist of items that should be included in reports of observational studies. [file MJA2-224-0-s001.pdf]

Supporting Information

Supplementary material

This appendix was part of the submitted manuscript and has been peer reviewed. It is posted as supplied by the authors.

Appendix to: Welsh J, Freeman-Robinson R, Butler DC, et al. Continuity of Care in General Practice in Australia: A Whole-Of-Population Serial Cross-Sectional Study. *Med J Aust* 2026; doi: 10.5694/mja2.70229.

Supplementary material: Continuity of care in general practice in Australia: a whole-of-population serial cross-sectional study

Table S1: MBS codes used to identify general practitioner visits

**All item numbers from following MBS groups and subgroups:**
A1, A2, A6, A7.12, A7.2, A7.4, A7.5, A7.6, A7.7, A7.8, A7.9, A7.10, A7.11, A11, A14, A15, A17, A18, A19, A20, A22, A23, A27, A30, A35, A36.1, A36.4, A40.1, A40.2, A40.10, A40.11, A40.12, A40.13, A40.15, A40.3, A40.14, A40.16, A40.19, A40.20, A40.21, A40.22, A40.25, A40.26, A40.27, A40.28, A40.30, A40.39, A40.40, A45.1, A45.2, A45.3
**Additional specific item numbers**
93287, 93288, 93300, 93303, 93291, 93292, 93301, 93302, 93304, 93305, 93306, 93307, 93308, 93309, 93310, 93311, 93400, 93401, 93402, 93403, 93404, 93405, 93406, 93407, 93408, 93409, 93410, 93411, 93421, 93422, 93423, 93431, 93432, 93433, 93434, 93435, 93436, 93437, 93438, 93439, 93440, 93441, 93442, 93451, 93452, 93453, 93469, 93470, 93475, 93479, 93715, 93716, 93717

Notes:

1. MBS=Medicare Benefits Schedule.
2. All items are those claimed by general practitioners (GP) or other medical practitioners (not including specialist or consultant physician) providing primary care services (out-of-hospital).
3. The definition of 'GP visit' is a unique combination of patient ID, date of service, and service provider ID, conditional on the MBS item number being for a GP visit. Under this definition, a patient who saw only one GP on a particular day had one visit, regardless of the number of MBS items claimed, while a patient who saw two (or more) different GPs on the same day had two (or more more) GP visits.
4. MBS group A44 was excluded because these pertained solely to delivery of COVID-19 vaccines and do not reflect usual general practice MBS services.

Table S2. Proportion (%) of the Medicare study population with at least 4 general practitioner visits (UPI coverage), 2016-17 to 2022-23.

	2016-17	2017-18	2018-19	2019-20	2020-21	2021-22	2022-23
Total	79.4	79.8	80.4	80.4	78.4	78.4	78.7
Sex							
Male	74.5	74.9	75.7	75.7	73.2	73.0	73.3
Female	84.2	84.5	85.0	84.9	83.4	83.6	83.8
Age group (years)							
0-14	70.3	70.3	71.1	69.9	64.3	64.9	65.1
15-24	73.7	73.9	74.5	74.1	71.5	70.6	70.2
25-44	77.1	77.6	78.4	78.6	76.9	76.3	76.3
45-69	85.3	85.6	86.0	86.2	85.7	85.5	85.6
≥70	94.6	94.8	95.0	95.1	95.2	95.3	95.2
Remoteness area							
Major cities	81.0	81.3	82.1	81.9	79.8	79.5	79.8
Inner regional	78.7	79.0	79.5	79.3	77.7	77.2	77.6
Outer regional	77.1	77.2	77.4	77.1	75.3	74.3	74.2
Remote	71.3	71.4	71.0	70.5	68.6	66.9	66.5
Very remote	67.6	68.2	68.1	67.3	65.7	64.4	64.6
Other	73.7	73.9	74.1	74.5	72.4	76.8	77.0
SEIFA IRSD							
Q1 (most disadvantaged)	82.6	82.5	82.7	82.2	80.0	79.3	79.4
Q 2	81.2	81.5	82.0	81.7	79.7	79.1	79.1
Q 3	80.2	80.6	81.3	81.1	79.2	78.7	78.8
Q 4	79.1	79.6	80.5	80.4	78.5	78.3	78.5
Q5 (least disadvantaged)	77.2	77.9	78.9	79.2	77.3	77.2	78.0
Missing	73.8	74.0	74.2	74.7	72.6	76.8	77.0

Notes:

1. UPI= Usual Provider Index. A UPI of 0.70 (or 0.75 or 0.80) means that at least 70% (or 75% or 80%) of general practitioner visits were provided by an individual's main (i.e. the same) provider.
2. Age at 1 January at the start of the 2-year period, ascertained from the Medicare Consumer Directory
3. Remoteness area is based on the Statistical Area Level 1 of the individual’s residence derived from the Medicare Consumer Directory. (https://www.abs.gov.au/statistics/standards/australian-statistical-geography-standard-asgs-edition-3)
4. Socio-Economic Indexes for Areas Index of Relative Socio-Economic Disadvantage (SEIFA IRSD) in population-based quintiles, from Q1 (most disadvantaged) to Q5 (least disadvantaged), based on the Statistical Area Level 1 information for the individual’s usual residence. (https://www.abs.gov.au/websitedbs/censushome.nsf/home/seifa)

Table S3. Proportion (%) of the eligible Medicare study population with high continuity of care using different UPI cut points, by patient characteristics 2016-17 to 2022-23.

	2016-17			2017-18			2018-19			2019-2020			2020-21			2021-22			2022-23		
	UPI ≥0.70	UPI ≥0.75	UPI ≥0.80	UPI ≥0.70	UPI ≥0.75	UPI ≥0.80	UPI ≥0.70	UPI ≥0.75	UPI ≥0.80	UPI ≥0.70	UPI ≥0.75	UPI ≥0.80	UPI ≥0.70	UPI ≥0.75	UPI ≥0.80	UPI ≥0.70	UPI ≥0.75	UPI ≥0.80	UPI ≥0.70	UPI ≥0.75	UPI ≥0.80
Total	32.1	28.1	22.3	31.7	27.7	21.9	31.3	27.3	21.5	33.8	29.6	23.5	37.2	32.8	26.3	35.7	31.3	25.0	35.5	31.1	24.7
Sex																					
Male	34.1	30.2	24.1	33.6	29.8	23.7	33.1	29.2	23.2	35.6	31.5	25.1	39.0	34.8	28.1	37.6	33.4	26.8	37.6	33.4	26.7
Female	30.4	26.3	20.7	30.0	25.9	20.3	29.7	25.6	20.0	32.3	28.0	22.0	35.7	31.1	24.8	34.1	29.5	23.4	33.8	29.2	23.0
Age group (years)																					
0-14	19.2	16.5	11.7	19.0	16.4	11.6	18.9	16.3	11.5	21.9	19.0	13.7	25.6	22.4	16.5	23.5	20.5	14.9	22.4	19.5	14.1
15-24	17.9	15.4	11.1	17.7	15.2	10.9	17.8	15.3	11.0	20.7	17.8	13.0	24.2	21.0	15.6	23.6	20.4	15.1	24.1	21.0	15.5
25-44	23.5	20.1	15.0	23.0	19.7	14.6	22.6	19.3	14.3	25.5	21.8	16.4	29.2	25.3	19.4	28.5	24.6	18.8	28.5	24.7	18.8
45-69	42.0	37.0	30.1	41.1	36.2	29.3	40.3	35.4	28.6	42.4	37.3	30.3	45.1	39.9	32.8	43.1	38.0	31.1	42.7	37.6	30.5
≥70	58.5	52.4	44.7	57.5	51.3	43.6	56.3	50.1	42.4	57.1	50.9	43.1	58.3	52.2	44.5	54.8	48.6	41.1	53.5	47.2	39.5
Remoteness area																					
Major cities	32.4	28.4	22.7	32.0	28.0	22.3	31.6	27.6	21.9	34.1	29.9	23.8	38.0	33.5	27.1	36.6	32.1	25.8	36.3	31.8	25.4
Inner regional	33.2	29.0	22.7	32.5	28.3	22.2	32.1	27.9	21.8	34.5	30.1	23.6	36.8	32.3	25.6	34.8	30.4	23.8	34.6	30.1	23.5
Outer regional	29.7	26.0	20.2	29.3	25.5	19.9	29.0	25.3	19.6	31.1	27.1	21.1	33.8	29.6	23.4	32.9	28.8	22.6	33.1	28.9	22.7
Remote	23.5	20.3	15.1	22.6	19.5	14.4	22.3	19.3	14.2	23.8	20.6	15.4	26.7	23.3	17.7	26.6	23.2	17.7	26.9	23.5	17.7
Very remote	14.1	11.9	8.1	14.4	12.2	8.5	14.1	11.9	8.2	15.2	13.0	9.2	16.7	14.4	10.4	16.2	13.9	10.0	15.3	13.1	9.2
Other	31.2	27.3	21.2	30.8	27.0	20.9	30.5	26.7	20.7	34.2	30.1	23.6	36.1	31.9	25.2	33.4	29.2	22.9	33.9	29.6	23.3
SEIFA IRSD																					
Q1 (most disadvantaged)	34.4	30.2	24.4	34.0	29.9	24.1	33.9	29.7	23.9	36.4	32.0	25.9	40.1	35.6	29.1	39.0	34.5	28.1	39.1	34.6	28.1
Q2	32.4	28.3	22.6	32.0	28.0	22.2	31.6	27.6	21.8	34.2	29.9	23.8	37.7	33.2	26.8	36.4	32.0	25.7	36.4	32.0	25.5
Q3	31.5	27.6	21.8	31.1	27.1	21.4	30.7	26.7	21.0	33.2	29.0	22.9	36.7	32.2	25.8	35.2	30.8	24.5	35.1	30.7	24.3
Q4	31.2	27.3	21.5	30.7	26.8	21.0	30.1	26.3	20.6	32.6	28.4	22.4	36.1	31.7	25.2	34.5	30.1	23.8	34.1	29.8	23.4
Q5 (least disadvantaged)	31.1	27.3	21.4	30.6	26.8	20.9	30.0	26.2	20.4	32.4	28.2	22.0	35.8	31.4	24.9	34.1	29.8	23.4	33.4	29.1	22.6
Missing	31.3	27.4	21.3	30.9	27.1	21.0	30.7	26.8	20.8	34.3	30.2	23.7	36.2	32.0	25.3	33.5	29.3	23.1	34.2	29.8	23.5
Number of general practitioner visits																					
4-6 visits	30.6	30.6	22.8	30.2	30.2	22.5	29.9	29.9	22.2	31.9	31.9	23.8	34.9	34.9	26.3	33.8	33.8	25.3	33.7	33.7	25.3
7-9 visits	29.7	24.9	18.2	29.3	24.5	17.9	29.0	24.2	17.6	31.4	26.3	19.2	34.6	29.3	21.7	33.4	28.1	20.6	33.3	28.0	20.5
10-14 visits	30.2	24.8	20.8	29.8	24.4	20.5	29.4	24.1	20.1	32.0	26.3	22.0	35.4	29.4	24.9	34.0	28.1	23.6	33.8	27.8	23.3
15-19 visits	31.3	26.5	22.1	30.8	26.1	21.7	30.4	25.7	21.2	33.1	28.0	23.3	36.5	31.2	26.2	34.7	29.5	24.7	34.6	29.3	24.4

20-39 visits	36.9	31.6	25.9	36.2	31.0	25.3	35.6	30.4	24.7	38.3	32.8	26.8	41.5	35.8	29.6	39.6	34.0	27.9	39.6	33.8	27.6
≥40	42.9	37.0	30.6	42.0	36.1	29.7	41.4	35.6	29.3	44.4	38.4	31.8	47.7	41.7	35.1	46.1	40.1	33.6	46.3	40.1	33.4

Notes:

1. Eligible population are those with ≥ 4 general practitioner visits in 2022-23.
2. UPI= Usual Provider Index. A UPI of 0.70 (or 0.75 or 0.80) means that at least 70% (or 75% or 80%) of general practitioner visits were provided by an individual's main (i.e. the same) provider.
3. Age at 1 January at the start of the 2-year period, ascertained from the Medicare Consumer Directory.
4. Remoteness area based on the Statistical Area Level 1 of the individual's residence. (https://www.abs.gov.au/statistics/standards/australian-statistical-geography-standard-asgs-edition-3)
5. Socio-Economic Indexes for Areas Index of Relative Socio-Economic Disadvantage (SEIFA IRSD) in population-based quintiles, based on the Statistical Area Level 1 of the individual's residence. (https://www.abs.gov.au/websitedbs/censushome.nsf/home/seifa)

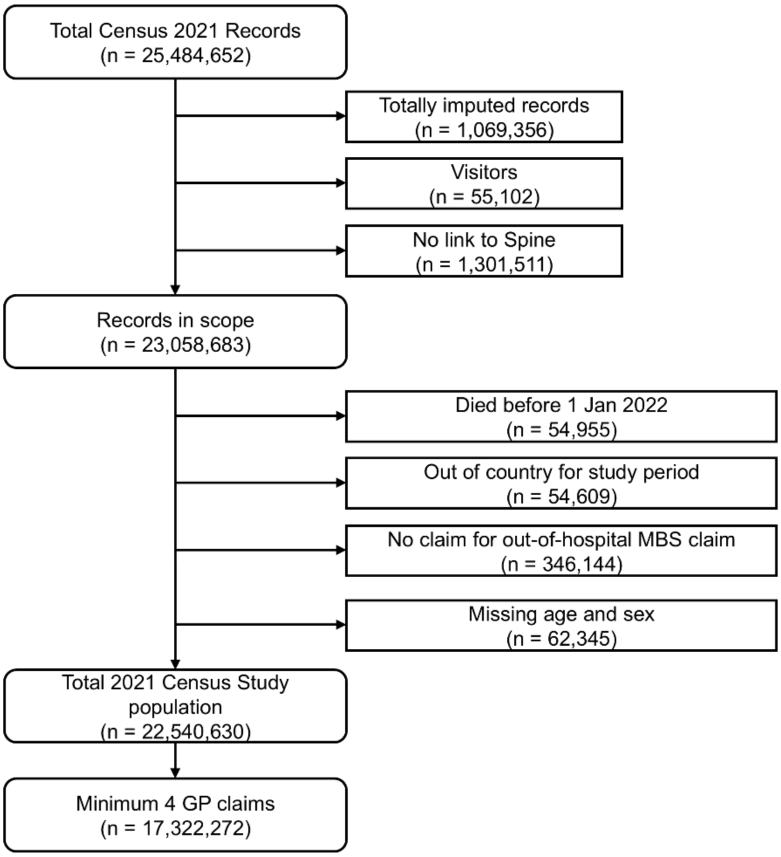

Figure S1. Study flow diagram for the Census study population for the 2022-23 study period.

STROBE Statement—checklist of items that should be included in reports of observational studies

	Item No	Recommendation	Page No
**Title and abstract**	1	(*a*) Indicate the study's design with a commonly used term in the title or the abstract	1
		(*b*) Provide in the abstract an informative and balanced summary of what was done and what was found	1
**Introduction**			
Background/rationale	2	Explain the scientific background and rationale for the investigation being reported	3
Objectives	3	State specific objectives, including any prespecified hypotheses	3
**Methods**			
Study design	4	Present key elements of study design early in the paper	4
Setting	5	Describe the setting, locations, and relevant dates, including periods of recruitment, exposure, follow-up, and data collection	4
Participants	6	(*a*) *Cohort study*—Give the eligibility criteria, and the sources and methods of selection of participants. Describe methods of follow-up<br>*Case-control study*—Give the eligibility criteria, and the sources and methods of case ascertainment and control selection. Give the rationale for the choice of cases and controls<br>*Cross-sectional study*—Give the eligibility criteria, and the sources and methods of selection of participants	4
		(*b*) *Cohort study*—For matched studies, give matching criteria and number of exposed and unexposed	

		Case-control study—For matched studies, give matching criteria and the number of controls per case	
Variables	7	Clearly define all outcomes, exposures, predictors, potential confounders, and effect modifiers. Give diagnostic criteria, if applicable	4
Data sources/ measurement	8*	For each variable of interest, give sources of data and details of methods of assessment (measurement). Describe comparability of assessment methods if there is more than one group	*4/13*
Bias	9	Describe any efforts to address potential sources of bias	4
Study size	10	Explain how the study size was arrived at	4
Quantitative variables	11	Explain how quantitative variables were handled in the analyses. If applicable, describe which groupings were chosen and why	4
Statistical methods	12	(*a*) Describe all statistical methods, including those used to control for confounding	4
		(*b*) Describe any methods used to examine subgroups and interactions	4
		(*c*) Explain how missing data were addressed	
		(*d*) *Cohort study*—If applicable, explain how loss to follow-up was addressed<br>*Case-control study*—If applicable, explain how matching of cases and controls was addressed<br>*Cross-sectional study*—If applicable, describe analytical methods taking account of sampling strategy	
		(*e*) Describe any sensitivity analyses	5

Continued on next page

Results

Participants	13*	(a) Report numbers of individuals at each stage of study—eg numbers potentially eligible, examined for eligibility, confirmed eligible, included in the study, completing follow-up, and analysed	5
		(b) Give reasons for non-participation at each stage	
		(c) Consider use of a flow diagram	
Descriptive data	14*	(a) Give characteristics of study participants (eg demographic, clinical, social) and information on exposures and potential confounders	Table 1
		(b) Indicate number of participants with missing data for each variable of interest	
		(c) *Cohort study*—Summarise follow-up time (eg, average and total amount)	
Outcome data	15*	*Cohort study*—Report numbers of outcome events or summary measures over time	
		Case-control study—Report numbers in each exposure category, or summary measures of exposure	
		Cross-sectional study—Report numbers of outcome events or summary measures	*Table 1, Figure 1*
Main results	16	(*a*) Give unadjusted estimates and, if applicable, confounder-adjusted estimates and their precision (eg, 95% confidence interval). Make clear which confounders were adjusted for and why they were included	5-6
		(*b*) Report category boundaries when continuous variables were categorized	
		(*c*) If relevant, consider translating estimates of relative risk into absolute risk for a meaningful time period	5-6
Other analyses	17	Report other analyses done—eg analyses of subgroups and interactions, and sensitivity analyses	

Discussion

Key results	18	Summarise key results with reference to study objectives	6
Limitations	19	Discuss limitations of the study, taking into account sources of potential bias or imprecision. Discuss both direction and magnitude of any potential bias	7-8

Interpretation	20	Give a cautious overall interpretation of results considering objectives, limitations, multiplicity of analyses, results from similar studies, and other relevant evidence	8
Generalisability	21	Discuss the generalisability (external validity) of the study results	8
**Other information**			
Funding	22	Give the source of funding and the role of the funders for the present study and, if applicable, for the original study on which the present article is based	

*Give information separately for cases and controls in case-control studies and, if applicable, for exposed and unexposed groups in cohort and cross-sectional studies.

Note: An Explanation and Elaboration article discusses each checklist item and gives methodological background and published examples of transparent reporting. The STROBE checklist is best used in conjunction with this article (freely available on the Web sites of PLoS Medicine at http://www.plosmedicine.org/, Annals of Internal Medicine at http://www.annals.org/, and Epidemiology at http://www.epidem.com/). Information on the STROBE Initiative is available at www.strobe-statement.org.
